# mRNA-Based Neoantigen Vaccines in Pancreatic Ductal Adenocarcinoma (PDAC)—A Promising Avenue in Cancer Immunotherapy

**DOI:** 10.3390/ijms262210988

**Published:** 2025-11-13

**Authors:** Jacek Kabut, Małgorzata Stopyra, Natalia Nafalska, Grzegorz J. Stępień, Michał Miciak, Marcin Jezierzański, Tomasz Furgoł, Krzysztof Feret, Iwona Gisterek-Grocholska

**Affiliations:** 1 Department of Oncology and Radiotherapy, Silesian Medical University, 40-514 Katowice, Poland; igisterek@sum.edu.pl; 2 Faculty of Medicine, Silesian Medical University, 41-800 Zabrze, Poland; s81569@365.sum.edu.pl (M.S.); s81185@365.sum.edu.pl (N.N.); s80961@365.sum.edu.pl (M.J.); s80882@365.sum.edu.pl (T.F.); 3 Faculty of Medicine, Medical University of Lodz, 90-419 Lodz, Poland; 4 Faculty of Medicine, Wroclaw Medical University, 50-367 Wroclaw, Poland; michal.miciak@student.umw.edu.pl; 5 Faculty of Medicine, Academy of Silesia, 40-555 Katowice, Poland; krzysztof.feret@akademiaslaska.pl

**Keywords:** pancreatic ductal adenocarcinoma (PDAC), immunotherapy, mRNA vaccines, tumor neoantigens, tumor microenvironment, immune checkpoint inhibitors, therapeutic resistance, personalized cancer treatment

## Abstract

Pancreatic ductal adenocarcinoma (PDAC) remains one of the most aggressive malignancies, with 5-year survival rates consistently below 5% despite advances in surgery, chemotherapy, and targeted therapy. Worldwide, PDAC remains highly lethal, with 458,918 new cases and 432,242 deaths in 2018—about a 94% mortality-to-incidence ratio. The limited therapeutic efficacy is largely attributed to the pronounced heterogeneity of the disease, late clinical presentation, and the strongly immunosuppressive tumor microenvironment. In recent years, mRNA-based vaccines encoding patient-specific neoantigens have emerged as a promising immunotherapeutic modality. By delivering tailored antigenic sequences, these vaccines are capable of eliciting potent cytotoxic T-cell responses against tumor-restricted epitopes, thereby enhancing tumor immunogenicity while minimizing off-target effects. This review summarizes the biological rationale underlying mRNA vaccination in PDAC, recent progress in preclinical and early clinical trials, and key obstacles related to antigen selection, delivery platforms, and the immunosuppressive stroma. The potential integration of neoantigen mRNA vaccines into multimodal therapeutic strategies, including immune checkpoint inhibition and chemotherapy, is also discussed, underscoring their prospective role in overcoming resistance mechanisms and improving clinical outcomes in PDAC. However, most current data come from early-phase trials, with long-term benefits yet unproven. Definitive conclusions on efficacy and survival await results from ongoing randomized studies expected by 2028–2029. Further progress in neoantigen identification, delivery systems, and combination strategies is crucial to fully harness mRNA vaccine potential in PDAC.

## 1. Introduction

### 1.1. Overview of Pancreatic Ductal Adenocarcinoma (PDAC)

Pancreatic ductal adenocarcinoma (PDAC) remains one of the most aggressive and lethal malignancies, characterized by an exceptionally poor prognosis and a mortality rate nearly equivalent to its incidence. Despite decades of research and clinical advancements, the five-year survival rate for patients diagnosed with PDAC remains below 5%, similar to statistics from thirty years ago [1]. According to Cancer Statistics 2021, the American Cancer Society reported approximately 60,430 new cases and 48,220 deaths for PDAC in the United States, ranking third after lung and bronchus cancer and colorectal cancer. In the 28 countries of the European Union, it was estimated that approximately 111,500 people (55,000 in males and 56,500 in females) will die from PDAC by 2025 [2,3]. Global Cancer Statistics 2018 showed that the incidence and mortality of PDAC were 458,918 and 432,242 in the world, respectively, and deaths account for about 94.2% of new cases [4,5].

Multiple determinants of PDAC risk have been delineated, encompassing demographic factors like male sex or black race, environmental and lifestyle exposures (e.g., any history of tobacco use, obesity), comorbid conditions (e.g., diabetes mellitus, chronic pancreatitis, *Helicobacter pylori* infection), and inherited susceptibility (e.g., pathogenic variants in hereditary pancreatic cancer genes and selected cancer predisposition syndromes). PDAC may also develop from a precancerous lesion of pancreatic intraepithelial neoplasia, a phenomenon often arising from acinar-to-ductal metaplasia, which can occur as a result of cellular injury or inflammation in the pancreas [1,6,7,8,9]. Distinct molecular features characterize early-onset PDAC: relative enrichment of *RAS* wild-type tumors versus the broader PDAC population, a higher prevalence of biallelic *CDKN2* inactivation via homozygous copy loss with overexpression of *FOXC2*—concordant with upregulation of *VIM*, *CDH11*, and *CDH2* that drive epithelial–mesenchymal transition—compared with cases of average onset (>55 years), and a lower frequency of *SMAD4* alterations than in late-onset disease (≥70 years) [2,3,4]. From a prevention and public-health standpoint, roughly one-third of the overall PDAC burden is attributable to the two-thirds of recognized modifiable lifestyle risks factors [10,11]. Many such exposures plausibly promote tumorigenesis through oxidative stress and chronic inflammation, and environmental pollutants have been associated with higher risks across multiple cancers, including PDAC [12,13]. Growing population-level exposure to these factors over recent decades may further amplify risk among genetically predisposed individuals. Nevertheless, PDAC etiology remains incompletely defined, underscoring the need for additional studies and evidence syntheses to refine risk stratification and optimize screening and primary prevention strategies [8,10,14,15].

### 1.2. Challenges in PDAC Treatment and Prognosis

**Figure 1:**
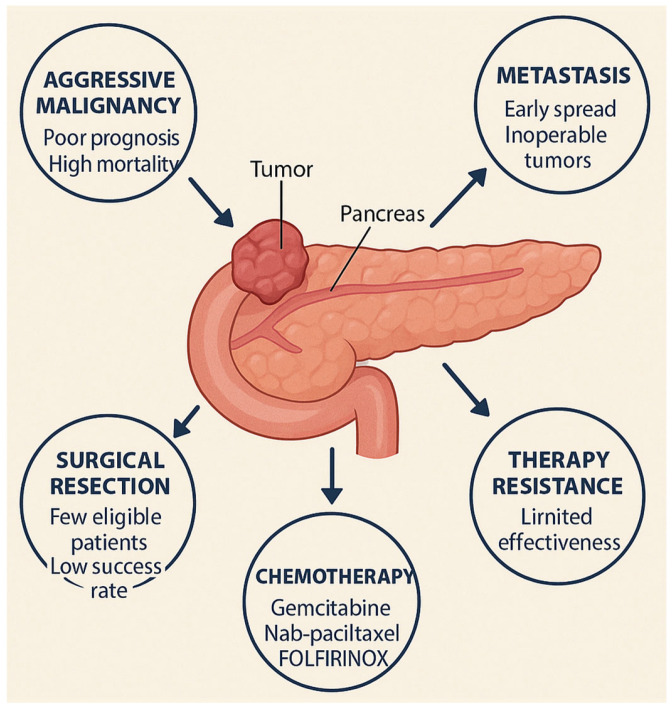
Clinical and biological barriers in pancreatic ductal adenocarcinoma (PDAC) management.

One of the primary factors contributing to the high mortality of PDAC is its anatomical location. Situated deep within the retroperitoneal space and surrounded by vital vascular structures and adjacent organs, pancreatic tumors are often challenging to resect, particularly in cases of local tissue infiltration. Furthermore, due to the central position of the pancreas and the nonspecific nature of early symptoms, diagnosis frequently occurs at an advanced stage of disease progression [10,14,16,17]. As a result, at the time of diagnosis, tumors commonly exhibit local invasion or distant metastases, rendering them inoperable [18,19]. Another key factor underlying the dismal prognosis of PDAC is its pronounced resistance to systemic therapies. This resistance is driven by multiple biological barriers that limit the efficacy of anticancer treatments (Figure 1). These barriers arise from three hallmark features of PDAC: a highly immunosuppressive tumor microenvironment (TME), pronounced genetic heterogeneity, and a propensity for early metastatic spread [16,20,21,22].

The current standard of PDAC therapy involves surgical resection combined with systemic chemotherapy. However, only approximately 20% of patients are eligible for surgery at the time of diagnosis due to advanced disease stage [7]. Even among those undergoing curative-intent resection, disease recurrence occurs in nearly 80% of cases within two years postoperatively [8]. For patients with unresectable, locally advanced, or metastatic PDAC, systemic chemotherapy regimens such as gemcitabine plus nab-paclitaxel or FOLFIRINOX (a combination of fluorouracil, oxaliplatin, irinotecan, and leucovorin) are commonly employed. While these regimens have modestly improved survival, their use is often limited by substantial toxicity, deterioration in quality of life, and the eventual emergence of drug resistance [9]. Alternative treatment approaches—including radiotherapy, targeted therapies, and immunotherapy—have also been investigated in this patient population; however, their clinical efficacy remains limited [2,3]. Given these challenges, PDAC continues to represent one of the most formidable obstacles in modern oncology, necessitating the development of more effective and durable therapeutic strategies.

### 1.3. Emerging Role of Immunotherapy in PDAC and Rationale for mRNA Vaccines

In light of the limited efficacy of currently available treatment modalities, there is a growing interest in innovative therapeutic strategies aimed at overcoming the inherent resistance mechanisms of PDAC. Among these, cancer vaccines have emerged as a promising approach, offering several advantages including multifaceted mechanisms of action, a favorable toxicity profile, broad therapeutic potential, and the ability to induce long-lasting immunological memory. A wide range of vaccine platforms have been evaluated in both preclinical and clinical settings, including peptide-based vaccines, dendritic cell-based vaccines, and viral vector platforms. Although encouraging results have been obtained in preclinical models, translating these findings into clinical efficacy remains a significant challenge. Recently, mRNA-based vaccines have garnered particular attention as a novel and versatile modality in cancer immunotherapy, including in the treatment of PDAC.

Thus, the aim of this review is to summarize the current state of knowledge and available evidence on the application of mRNA vaccines in the PDAC therapy.

## 2. Landscape of mRNA-Based Cancer Vaccines Research in PDAC

### 2.1. Molecular and Immunological Landscape of PDAC

PDAC is distinguished by explicit molecular heterogeneity and an immunosuppressive TME. Genomic analyses have defined distinct PDAC subtypes (e.g., classical versus basal-like) with divergent biology, reflecting the extensive inter- and intratumoral molecular variability that complicates efficient treatment [4,21]. A determinative feature of PDAC is a dense desmoplastic stroma, rich in cancer-associated fibroblasts and extracellular matrix, which creates a physical barrier to infiltration of antitumoral immune cells [22,23]. Moreover, this fibrotic and hypovascular microenvironment promotes tumor hypoxia and limits drug delivery, while also actively contributing to immune evasion. Tumor and stromal cells engage in aberrant signaling that shifts the immunoregulatory balance toward a pro-tumor state [21]. PDAC is often termed a “cold” tumor, as they exhibit low immunogenicity and unfavorable miroenvironment [4,23,24].

Instead, immunosuppressive populations predominate—including tumor-associated macrophages (TAMs) polarized to an M2 phenotype, myeloid-derived suppressor cells (MDSCs), and regulatory T cells (Tregs)—all of which secrete factors that inhibit effector T-cell function [22,25]. Elevated levels of anti-inflammatory cytokines such as transforming growth factor-β (TGF-β) and interleukin-10 (IL-10), as well as pro-angiogenic vascular endothelial growth factor (VEGF) are prevalent among PDAC, further reinforcing immune tolerance [23]. PDAC cells and infiltrating immune cells may express and upregulate multiple immune checkpoint molecules (e.g., PD-L1 and PD-1, CTLA-4, TIM-3, LAG-3, and VISTA) that inhibit antitumoral T-cell activation in the TME. These intrinsic and extrinsic mechanisms of immunosuppression underlie PDAC’s notorious resistance to immunotherapy [26,27]. Notably, single-agent checkpoint inhibitors (e.g., anti-PD-1) have shown minimal efficacy in PDAC, largely due to the paucity of pre-existing T-cell infiltration and the multitude of active, parallel immunosuppressive pathways. Overall, the hostile immune landscape—combining pronounced tumor heterogeneity, a barrier-rich stroma, and active immune suppression—presents a major hurdle to conventional therapies. This context provides a strong rationale for novel immunotherapeutic approaches. By understanding and eventually modulating the PDAC microenvironment, mRNA vaccine strategies aim to overcome these barriers [23].

### 2.2. Tumor Antigens and Neoantigens in PDAC

Effective cancer vaccines depend on identifying appropriate antigenic targets expressed by tumor cells. These targets belong to two broad categories: tumor-associated antigens (TAAs) and tumor-specific antigens (TSAs). TAAs are proteins that are expressed in healthy tissue, however also aberrantly present in cancer [4]. In PDAC they include, e.g., mucin-1 (MUC1), carcinoembryonic antigen (CEA), and glypican-1 (GPC1) [12,13,28,29]. For instance, GPC1 is frequently overexpressed in PDAC and has been correlated with poor prognosis and contributing to pro-tumor state [30]. While the presence of TAAs on tumor cells can be recognized by the immune system, their expression in healthy tissue raises concerns of on-target/off-tumor effects and induction of self-tolerance that may limit vaccine potency [4,31]. By contrast, TSAs, also called neoantigenes, are molecules arising from somatic mutations exclusive to cancer. Actually, there is no expression of them on healthy cells, making these targets highly attractive. However, TSAs are rather patient-specific and result from the individual’s unique mutational signatures, meaning the landscape of suitable neoepitopes can vastly differ from case to case [32].

The process of neoantigen identification begins with the next-generation sequencing of tumor tissue. Computational analysis is then employed to predict which mutations are likely to be recognized by the patient’s immune system. Key steps include searching for mutations that alter protein coding, assessing peptide binding affinity to HLA class I and II (using tools such as NetMHCpan and MHCflurry), and evaluating stability of the peptide–MHC (major histocompatibility complex) molecules. High transcript expression of the mutant gene can be an important criterion, as abundant expression may compensate for moderate HLA binding affinity and increase the chance of immunogenic T-cell responses. Candidate neoantigens are typically validated through in vitro assays—for example, by assessing the ability of mutant peptides to be recognized by the patient’s T cells or by mass spectrometry-based immunopeptidomics to confirm their natural presentation on tumor HLA molecules [33,34,35]. However, experimental validation at large scale remains a challenge, mainly due to the costs and limited number of patient samples, which restricts the amount of neoantigens that can be tested.

PDAC typically exhibits a relatively low tumor mutational burden, and thus there remains a limited number of potentially targeted neoantigens, especially compared to malignancies like melanoma or lung cancer. Indeed, around 1% of PDAC patients harbor high tumor mutational burden, mismatch-repair deficiency and high microsatellite instability, and are more prone to response to checkpoint inhibition, whereas the majority of PDAC patients remain unresponsive [36,37]. Tumor heterogeneity in PDAC adds another layer of complexity to antigen selection. Different subclones within the same tumor may carry distinct mutations, meaning an immunogenic neoepitope from one region of the cancer might not be ubiquitous among all tumor cells [4]. Consequently, vaccines must target a broad panel of neoantigens to cover the clonal diversity of a patient’s tumor. Indeed, the trials we have discussed in Section 3. encoded as many as 20 different neoantigens per patient in an effort to maximize coverage. However, despite these challenges, our ability to predict and validate true neoantigens improves and the hope is that mRNA-based vaccines encoding patient-specific neoepitopes will elicit robust T cells that recognize and destroy PDAC cells while sparing normal tissue.

### 2.3. mRNA Vaccine Platforms: Design, Formulation, and Delivery

mRNA vaccine technology offers a flexible and powerful platform for presenting antigens, but its success relies on sophisticated molecular design and accurate delivery methods. Broadly, mRNA cancer vaccines come in two forms: conventional (non-replicating) mRNA and self-amplifying mRNA (saRNA). Conventional mRNAs encode only the antigen of interest and typically include regulatory elements such as a 5′ cap, 5′ and 3′ untranslated regions (UTRs), and a poly(A) tail, all optimized for stability and efficient translation. Self-amplifying mRNAs, by contrast, are larger constructs that encode not only the target antigen but also viral replicase proteins (commonly derived from alphaviruses) that enable the RNA to autonomously replicate in the cytoplasm [38,39,40]. By producing multiple copies of themselves in situ, saRNA vaccines can greatly amplify antigen expression. Preclinical studies have shown that saRNA platforms can achieve equivalent immune responses at lower doses compared to conventional mRNA vaccines. Of course, this feature has implications for manufacturing and cost [41,42]. On the other hand, the larger size of replicating mRNAs may pose challenges for delivery and can overly activate innate immune responses, which must be carefully managed [39].

Regardless of the mRNA type, a number of structural optimizations are applied to enhance vaccine performance. Codon optimization of the open reading frame (ORF) is routinely performed to match human tRNA abundance and avoid rare codons, thereby improving translation efficiency. Similarly, the UTRs are engineered to stabilize the mRNA and facilitate ribosome recruitment. For example, the poly(A) tail, human β-globin 5′ and 3′ UTRs are often used for their known contribution to mRNA stability and translational efficiency [43]. Chemically modified nucleosides represent another novel innovation: substituting uridine with pseudouridine or 1-methylpseudouridine in the mRNA can significantly reduce activation of pattern recognition receptors (like TLR7/8, RIG-I) that could trigger innate responses and inhibit protein translation [44]. The described modifications enable mRNA vaccines to avoid the paradox of self-sabotaging their own expression. At the same time, a certain degree of innate immune stimulation is desirable to act as a built-in adjuvant. An elegant approach to achieve this balance has been the development of TriMix: a cocktail of mRNAs encoding three immune-stimulatory proteins (CD70, CD40L, and constitutively active TLR4). When co-delivered with an antigen mRNA, TriMix provides maturation signals to dendritic cells and co-stimulation for T cells, thereby enhancing vaccine-induced immunity [40,45]. This strategy highlights the versatility of mRNA platforms—multiple mRNAs can be combined to simultaneously encode antigens and adjuvant signals.

**Figure 2:**
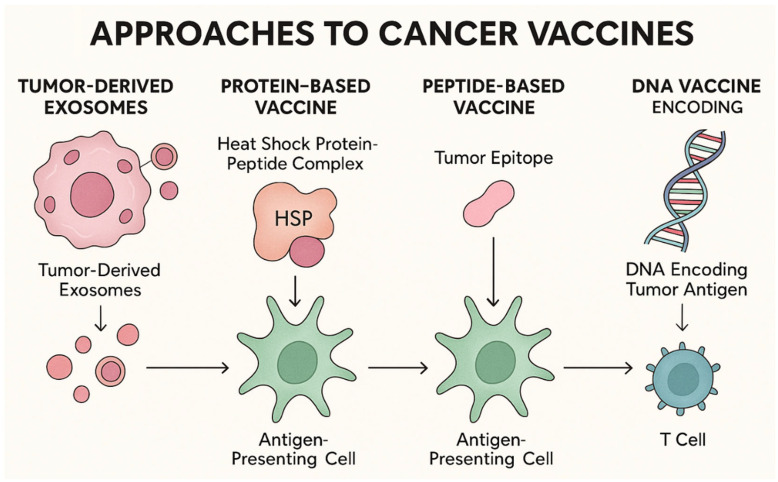
Alternative cancer vaccine platforms and their mechanisms of action.

Naked mRNA is susceptible to rapid degradation by RNases and struggles to cross cell membranes due to its large size and polyanionic charge [46,47]. The advent of nanoparticle carriers has largely solved this issue. Lipid nanoparticles (LNPs) have become the leading system for mRNA delivery. They typically consist of an ionizable lipid (crucial for cellular intake and facilitates endosomal escape), non-cationic phospholipids, cholesterol to provide stability, and a polyethylene glycol-lipid to prolong circulation and storage [48,49,50]. LNPs form particles capable of protecting mRNA and efficiently fusing with cell membranes to deliver the mRNA into the cytosol. These carriers facilitate efficient transport of mRNA into immune cells, particularly dendritic cells, which subsequently present the translated antigens on their surface via MHCs—an essential step for initiating adaptive immune responses [48,51]. Moreover, the incorporation of adjuvants that stimulate immune activation has been shown to further enhance the immunogenicity and therapeutic efficacy of mRNA vaccines, as demonstrated in multiple preclinical and clinical studies [52,53]. Alternative delivery systems under exploration include polyplexes (polymers that bind and condense mRNA), peptide-based nanoparticles, and nanoemulsions [54,55]. In addition, chemical modifications of mRNA—such as the incorporation of modified nucleotides—can improve its stability and bioavailability, leading to more sustained and efficient antigen expression in vivo. These strategies have been shown to enhance cytotoxic T cell responses and antibody production, ultimately promoting the effective elimination of tumor cells [56]. Another promising strategy involves the use of tumor-derived exosomes (TEXs)—membrane-bound vesicles released by tumor cells that contain tumor-associated antigens and adhesion molecules [57,58]. Protein-based vaccines, particularly those utilizing heat shock protein-peptide complexes (e.g., HSPPC-96), carry tumor-derived peptides and activate the immune system through antigen presentation by antigen presenting cells (APCs) [59]. Peptide-based cancer vaccines are designed to target specific tumor epitopes—such as *KRAS*, telomerase, or survivin—yet their efficacy is often limited by MHC restriction and the typically monoclonal nature of the T cell response [60]. DNA vaccines, which encode selected tumor antigens (e.g., MUC1, survivin, ENO1, VEGFR-2), can induce cytotoxic responses through endogenous antigen expression in host cells. However, their clinical application is constrained by safety concerns, including the risk of anti-DNA antibody induction and potential genomic integration [4]. Approaches to cancer vaccines are summarized in Figure 2.

Finally, the route of administration can influence vaccine performance and there is a wide range of available approaches: from intradermal and intramuscular—the most common routes, to more localized, such as intratumoral injections [61]. In summary, due to improvement of nucleic acid engineering and development of advanced delivery systems, mRNA vaccines have overcome many initial limitations (like unacceptable instability or low in vivo potency), however further research is needed to fully establish their clinical translation in the treatment of PDAC.

### 2.4. Comparison with Other Vaccine Platforms

Numerous vaccine modalities have been explored in PDAC over the last decades, including those based on peptides, viral vectors, dendritic cells, or exosomes. While some of them have shown immunogenicity in preclinical or early clinical studies, their overall therapeutic impact in PDAC has been limited, highlighting the need for more advanced and improved approaches [4]. Peptide-based vaccines typically involve one or several short peptides derived from TAAs or patient-specific neoantigens, administered with an immunologic adjuvant (such as GM-CSF or Toll-like receptor agonists) [62,63]. These vaccines are relatively easy to manufacture and well-tolerated, but they often elicit only modest T-cell responses. One issue is that short peptides usually trigger predominantly CD8^+^ T-cell responses restricted to a particular HLA allele, which limits their full potential [64]. Additionally, because TAAs are self-antigens, activity of peptide vaccines can be hindered by immune tolerance [65]. In PDAC, peptide vaccines against common antigens like telomerase, or MUC1 have demonstrated safety and immune activation, but no significant improvement in survival as monotherapies has been observed [66,67].

DNA vaccines (plasmid DNA encoding a tumor antigen) have also been tested in PDAC treatment. One of the examples is a plasmid vaccine encoding enolase-1 (ENO1), an antigen overexpressed in PDAC, which has successfully increased survival in a mice model [68]. DNA vaccines are inexpensive and thermostable, and they can drive endogenous antigen production like mRNA does. However, DNA must enter the cell nucleus to be further transcribed, which might be inefficient in vivo; methods like electroporation are often required to improve DNA uptake by cells [69,70]. More importantly, there are safety concerns regarding DNA vaccines, including the risk of genomic integration or development of autoimmune reactions [69].

Viral vector vaccines, using platforms such as adenovirus or poxvirus engineered to express antigens, have elicited antitumoral immunogenicity in some cancers [71]. One notable example is GVAX (an irradiated, GM-CSF-secreting allogeneic pancreatic tumor cell vaccine) used in combination with a Listeria monocytogenes vector expressing mesothelin (CRS-207). This modality has improved survival of metastatic PDAC patients and shown minimal toxicity [72]. Viral vectors can induce strong T-cell responses, however pre-existing anti-vector immunity (against common viruses like adenovirus) may limit the widespread application of these vaccines [73].

Dendritic cell (DC) vaccines involve autologous dendritic cells loaded with tumor antigens. They can also be personalized and have shown some success in cancer treatment. However, the designing process is labor-intensive and patient-specific, and the immunosuppressive microenvironment of PDAC can impair the function of DCs [74,75,76]. Still, a few trials (e.g., using DCs loaded with MUC1 peptides) have reported vaccine-induced T-cell responses in PDAC, though without remarkable clinical outcomes [77].

Compared to above-mentioned platforms, mRNA vaccines offer compelling advantages. They do not integrate with the host genome and therefore carry no risk of genomic modifications, highlighting their distinguished safety profile [61,78]. They can be produced rapidly, which is ideal for large-scale personalized neoantigen vaccine distribution [79]. Moreover, a single mRNA vaccine can encode numerous epitopes (even entire sets of neoantigens or multiple TAAs), providing a multi-targeted attack in one product. mRNA vaccines inherently engage both the CD8^+^ and CD4^+^ T-cells [53,80,81]. Finally, unlike protein or peptide vaccines which may be limited by HLA specificity, mRNA vaccines may elicit broader immune responses [82]. Taken together, these features explain the rising enthusiasm for mRNA vaccines over more traditional cancer vaccine approaches [78].

### 2.5. Mechanism of Action

mRNA vaccines trigger both humoral and cytotoxic immune responses against PDAC cells. The core principle of mRNA-based cancer vaccines lies in their capacity to direct the immune system toward TAAs. By introducing mRNA that encodes these antigens, the vaccines are designed to generate a focused immune reaction against cancerous cells while minimizing damage to normal tissues [4]. Once administered, the mRNA is internalized by APCs—especially DCs—where it is translated into the corresponding antigenic proteins. After endocytosis, LNPs release the mRNA into the cytoplasm of APCs, allowing translation into TAAs and activating antigen processing and presentation pathways that promote an anticancer immune response [11,83].

After mRNA escapes the endosome, ribosomes begin translating it into proteins, which are subsequently processed by the ubiquitin–proteasome system to generate peptides suitable for antigen presentation. These peptide fragments are then transported via secretory vesicles to the endoplasmic reticulum, where they are loaded onto MHC class I and II molecules and displayed on the surface of APCs. This process leads to T-cell activation. Cytotoxic CD8+ T cells (Tc) recognize antigens presented by MHC class I, while MHC class II molecules activate helper CD4+ T cells (Th). Through T-cell receptors (TCRs), T cells identify these antigens and perform multiple interactions between immune checkpoint molecules on APCs and lymphocytes. The immune response is further strengthened by pro-inflammatory cytokines such as IL-1, IL-2, and IL-12, which support T-cell proliferation and effector differentiation. Activated CD8+ T cells release granzymes, perforin, TNF-α, and interferon-γ, promoting inflammation and inducing apoptosis in neoplastic cells [84,85].

**Figure 3:**
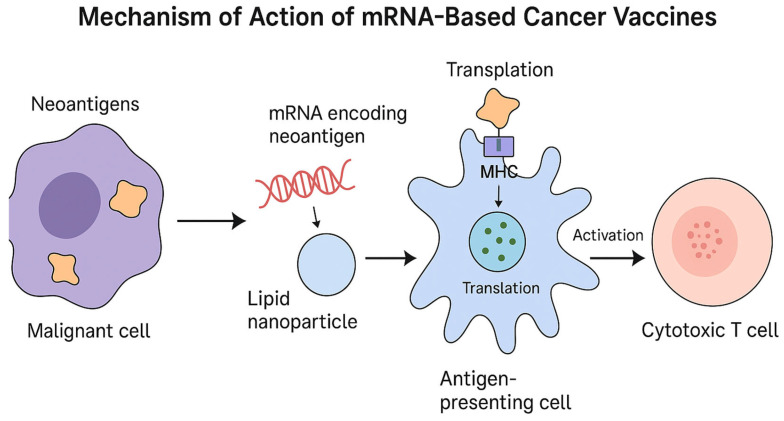
Mechanism of action of mRNA-based cancer vaccines.

The antigens can also stimulate B cells, which, with the help of CD4+ T cells, differentiate into plasma cells that secrete antigen-specific antibodies. This humoral immune response works synergistically with cytotoxic CD8+ T-cell activity to enhance the destruction of cancer cells. Additionally, the engagement of co-stimulatory immune checkpoints further drives the activation of antigen-specific T cells, while B lymphocytes are simultaneously induced to produce anticancer antibodies [86,87]. The mechanism of mRNA vaccines in PDAC is similar to the general mode of action observed for mRNA-based vaccines. Figure 3 illustrates a schematic overview of these activities.

### 2.6. Combination Strategies to Overcome Immune Resistance

Given the immunosuppressive features of PDAC, it seems inevitable that combination strategies, rather than mRNA vaccines alone will be required to unleash the full potential of mRNA vaccines [4]. Therapeutic cancer vaccines aim to generate a pool of tumor-specific T cells, however their efficacy in monotherapy may be blunted by checkpoints and suppressive cells in the TME. Combining mRNA vaccines with immune checkpoint inhibitors (ICIs) is a particularly compelling approach. ICIs such as anti-PD-1, anti-PD-L1, or anti-CTLA-4 can prevent or reverse T-cell inhibition, thereby amplifying the function of vaccine-induced T cells [88]. Vaccines prime critical antigen-specific T-cells, while ICIs sustain the effector function of those cells, by modulating a “cold” tumor—a clear synergistic interaction [4]. Checkpoint blockade then keeps those T cells active upon encountering tumor antigen. Consistent with this rationale, ongoing trials in PDAC are now evaluating mRNA neoantigen vaccines in combination with PD-1/PD-L1 inhibitors. A first-in-human study of a personalized mRNA neoantigen vaccine (autogene cevumeran, by BioNTech) combined with atezolizumab (anti–PD-L1 antibody) and chemotherapy in patients with resectable PDAC has reported promising results. 50% of vaccinated patients have developed neoantigen-specific T-cell responses, and those responders experienced a longer median recurrence-free survival compared to non-responders. While the number of participants was small (n = 16), this finding has confirmed that a mRNA neoantigen vaccine can result in improved outcomes when appropriately supported by other therapies [4,89].

Combining immunotherapy with chemotherapy may be advantageous, as certain chemotherapeutics (like gemcitabine or cyclophosphamide) can regulate MDSCs or Tregs, thereby decreasing immunosuppressive tendencies [90]. Standard PDAC chemotherapy regimens such as FOLFIRINOX can induce immunogenic cell death, releasing tumor antigens and damage-associated molecular patterns (DAMPs), which enhances and promotes the expansion of cancer-targeting T cells [91]. Indeed, the autogene cevumeran trial mentioned above included chemotherapy, and it seems possible that cytotoxic therapy had an impact on altering the TME in ways that synergized with the autogene cevumeran vaccine.

Another combination strategy is stromal remodeling. Agents targeting the desmoplastic stroma or cytokines might improve lymphocyte infiltration. For instance, inhibitors of the TGF-β pathway, which is a key driver of fibrosis and immune evasion in PDAC, could be combined with a vaccine to enhance T-cell penetration [92]. Enzymatic degradation of stromal barriers (e.g., PEGylated hyaluronidase against hyaluronic acid in the matrix) is another approach that has shown promising results in preclinical studies and could logically be extended to vaccine modalities [93]. Leveraging the mRNA technology, some approaches employ multiple therapeutic modalities into one vaccine. For example, an mRNA mixture encoding immune modulators can impact the TME. A recent example is mRNA-2752, an intratumorally administered mRNA vaccine encoding the T-cell stimulator OX40L and pro-inflammatory cytokines IL-23 and IL-36γ. This therapy has shown the activation of dendritic cells and CD8+ T cells locally and has been tested alone or combined with durvalumab in patients with solid tumors [94,95].

Therefore, mRNA vaccines, especially when combined with synergistic immunotherapies, have a potential to break through PDAC’s prominent resistance mechanisms. Ongoing clinical research is focused not only on potential drug combinations but also on identifying predictive biomarkers that could find patients who are most likely to benefit from a mRNA vaccine combined with another medication [4]. In conclusion, while mRNA-based neoantigen vaccines offer a highly personalized approach against PDAC, their greatest potential likely lies in integration into combination therapy. With several clinical trials assessing mRNA vaccine combinations in PDAC, the coming years will shed light on their avenue to overcome tumor-induced immunosuppression and improve patient outcomes.

## 3. Current Evidence of mRNA-Based Vaccines in the PDAC Therapy

One of the first studies demonstrating the clinical potential of personalized mRNA-based cancer vaccines in patients with PDAC was conducted by Rojas et al., inspired by the success of mRNA vaccines against SARS-CoV-2 [89]. A major challenge in developing effective cancer vaccines remains the induction of a durable and functional T cell response specifically targeting tumor antigens.

The study evaluated mRNA-lipoplex vaccines designed individually for each patient based on tumor-specific neoantigens arising from somatic mutations. In this phase I trial with extended follow-up (median: 3.2 years), patients received multimodal therapy comprising surgical resection, the PD-L1 checkpoint inhibitor atezolizumab, the personalized mRNA vaccine autogene cevumeran (encoding selected neoantigens in uridine-containing optimized mRNA-lipoplexes), and a modified FOLFIRINOX regimen. A significant improvement in relapse-free survival (RFS) was observed: 75% at 3 years among vaccine responders compared to 12.5% in non-responders (HR = 0.14; *p* = 0.007), indicating a clinically meaningful benefit associated with vaccine-induced immune responses [89,96].

At an extended median follow-up of 3.2 years from a phase I post-surgical trial, the authors found that patients who developed vaccine-induced T-cell responses (n = 8) exhibited significantly longer RFS compared with non-responders lacking such T-cell induction (n = 8; median RFS 13.4 months; *p* = 0.007). Patients demonstrating immunological responses to autogene cevumeran exhibited robust induction of neoantigen-specific CD8+ T cell clones with an estimated mean survival duration of 7.7 years (range: 1.5 to roughly 100 years). Approximately 20% of these clones were predicted to persist for several decades—exceeding expected patient lifespan. In 86% of vaccinated individuals, these CD8+ T cell clones remained detectable up to three years post-vaccination, including high-affinity clones directed against PDAC-specific neo-epitopes. PhenoTrack analysis revealed that 98% of vaccine-induced clones were absent in pre-immunization tumor samples and acquired a cytotoxic tissue-resident memory (TRM) phenotype with preserved effector function. In two vaccine responders, disease recurrence was associated with a decline in vaccine-induced CD8+ T cells and loss of neoantigen expression in recurrent tumors, suggesting immune editing and selective elimination of antigen-expressing cancer cells [97]. In summary, the autogene cevumeran vaccine elicited robust, durable, and functionally competent neoantigen-specific CD8+ T cell responses in PDAC patients, potentially contributing to prolonged RFS. These findings support the promise of mRNA-lipoplex vaccines targeting individualized neoantigens as a strategy to overcome key barriers in cancer vaccine development [96,97].

The encouraging results from the phase I study served as the basis for the launch of a multicenter phase II clinical trial—IMCODE003 (NCT05968326)—aimed at evaluating the efficacy and safety of autogene cevumeran in combination with atezolizumab and modified FOLFIRINOX (mFFX), compared to mFFX alone, in patients who have undergone pancreatoduodenectomy. The trial is expected to enroll 260 participants, with study completion and results anticipated by 2029 [98].

In another phase I clinical trial (NCT03289962), conducted by Lopez et al., the safety, tolerability, and immunogenicity of the individualized mRNA vaccine autogene cevumeran were evaluated. This vaccine was designed based on the somatic mutational landscape of each patient’s tumor and targeted up to 20 selected neoantigens. It was administered either as monotherapy (n = 30) or in combination with the PD-L1 checkpoint inhibitor atezolizumab (n = 183) in patients with advanced solid tumors, including pancreatic cancer. Interim analysis demonstrated favorable tolerability and immunogenicity, with vaccine-induced immune responses observed in 71% of patients, involving CD4+ and/or CD8+ T cells. These responses were detectable for up to 23 months following treatment initiation. Neoantigen-specific CD8+ T cells constituted a median of 7.3% of circulating CD8+ T cells, with intratumoral levels reaching up to 7.2% of tumor-infiltrating lymphocytes. Clinical responses were reported in one patient treated with monotherapy and in two patients receiving combination therapy, despite the presence of unfavorable predictive factors for immunotherapy response. These findings support the potential of personalized mRNA vaccines as an effective immunotherapeutic approach, particularly in the early treatment setting of malignant tumors [99,100].

The phase I clinical trial (NCT03468244), conducted at Changhai Hospital in Shanghai, aimed to assess the safety, tolerability, and efficacy of a personalized mRNA vaccine encoding neoantigens in patients with advanced gastrointestinal cancers, including PDAC. The intervention involved the administration of up to 20 synthetic long peptides encoding patient-specific neoantigen sequences in the form of an mRNA vaccine, delivered in parallel with standard therapy in accordance with National Comprehensive Cancer Network guidelines. To date, no detailed data have been published regarding the efficacy or safety of the neoantigen-encoding mRNA vaccine specifically in pancreatic cancer patients enrolled in this trial. However, preliminary results in patients with other gastrointestinal malignancies, such as esophageal squamous cell carcinoma, suggest potential therapeutic benefit of this strategy [101].

Another clinical study (NCT03948763), for which detailed results have not yet been published, aimed to assess the safety, tolerability, and preliminary efficacy of the mRNA vaccine mRNA-5671/V941 (V941), administered as monotherapy or in combination with pembrolizumab in 70 patients with advanced solid tumors harboring *KRAS* mutations (G12D, G12V, G13D, G12C), including PDAC. The treatment was generally well tolerated, with no serious adverse events attributed to the vaccine. While immune responses were observed, detailed characterization of these responses and their clinical relevance remains pending [102].

A separate phase I trial (NCT05916261), initiated in April 2023, is currently evaluating the safety, tolerability, and preliminary efficacy of the personalized mRNA-0217/S001 vaccine encoding tumor-specific neoantigens, administered either alone or in combination with pembrolizumab (an anti–PD-1 immune checkpoint inhibitor) in patients (n = 54) with advanced PDAC [103].

An additional early-phase study (NCT06156267), launched in January 2024 by Fudan University in collaboration with Shanghai Regenelead Therapies Co., is currently pending recruitment. The study aims to enroll 30 participants and will assess the safety, tolerability, and preliminary efficacy of a personalized mRNA vaccine encoding neoantigens administered in combination with adebrelimab, a PD-L1 checkpoint inhibitor, in patients with advanced PDAC [104].

The clinical trial NCT06353646 is currently evaluating the safety and efficacy of the cancer vaccine XH001, which targets tumor-specific neoantigens and is administered sequentially with ipilimumab (an anti–CTLA-4 monoclonal antibody) and chemotherapy in patients with PDAC following radical surgical resection. Another ongoing study, NCT06496373, is investigating the XP-004 mRNA-based vaccine encoding personalized neoantigens in combination with a PD-1 inhibitor as adjuvant therapy in patients with advanced solid tumors, including post-operative PDAC. Both trials are currently recruiting participants, with completion expected in 2026–2027 [105,106].

The summary of discussed clinical trials is presented in Table 1.

## 4. Summary, Limitations, and Future Perspectives

This article was meant to provide a multidimensional overview of PDAC biology and the underlying principles of mRNA vaccine technology. PDAC remains one of the most lethal malignancies, characterized by a high recurrence rate, limited efficacy of systemic therapies, and profound biological resistance. These unfavorable features are largely attributable to the tumor’s immunosuppressive microenvironment, pronounced genetic heterogeneity, and early metastatic potential. Despite the development of multimodal treatment approaches—including cytotoxic chemotherapy and molecularly targeted therapies—long-term survival outcomes for patients with PDAC remain dismal. This underscores the urgent need to explore and implement novel, more effective therapeutic strategies.

Broad implementation of mRNA vaccines into routine treatment modalities may pose several challenges related to the distinctive biology of PDAC. One of the major limitations of this approach is that only a minority of patients—approximately 20%—present with resectable disease at diagnosis. This highlights the need for further studies evaluating the efficacy of neoantigen-targeted mRNA vaccines in patients with borderline resectable, locally advanced, or metastatic PDAC. Integration of such vaccines into multimodal treatment regimens that include local therapies, chemotherapy, and immunotherapy may extend their applicability to a broader patient population. As discussed, molecular heterogeneity may restrict the identification of adequate targets for mRNA vaccines, namely TSAs that cover the full landscape of the tumor. Additionally, effective delivery of mRNA and robust responses to immunotherapy may be limited by an unfavorable pro-tumoral microenvironment. Notably, domination of immunosuppressive immune cells and the activation of anti-inflammatory molecules constitute serious obstacles to the success of immunotherapy in PDAC. Furthermore, dysregulation of immune checkpoints may inhibit immune responses, thereby reducing the efficiency of antitumoral vaccines. It seems crucial to further explore the biology and distinctiveness of pancreatic cancer at the basic science level, as well as the application of immune checkpoint inhibitors, in combination with mRNA vaccines, in treatment regimens.

Another important factor limiting the full potential of mRNA vaccines in the treatment of PDAC is the instability of mRNA and its susceptibility to enzymatic degradation, as well as poor vascularization within the TME. It could be addressed by extensive research and development of next-generation delivery platforms. The personalized nature and high costs of manufacturing mRNA vaccines represent additional obstacles. At the preclinical stage, designing vaccines and conducting comprehensive studies on targets involve highly qualified research backgrounds. In addition, next-generation sequencing, required for target identification, remains a relatively expensive technology, thereby limiting large-scale application. Moreover, consistent quality control and standardization necessitates close and well-structured cooperation between hospitals, regulatory agencies, and industry.

Complementarily, the results from recent clinical trials on the safety and efficacy of mRNA vaccines in PDAC treatment were discussed. Early-phase clinical trials (phase I/II) have demonstrated the feasibility, safety, and immunogenicity of these vaccines, particularly in patients with resectable PDAC. Notably, vaccine-induced neoantigen-specific CD8^+^ T cells with sustained effector function and long-term persistence were observed in responders, correlating with prolonged relapse-free survival. When administered in combination with immune checkpoint inhibitors and cytotoxic chemotherapy, these vaccines have shown potential to enhance antitumor immune responses and improve clinical outcomes. However, research gaps and issues regarding the implementation of this novel technology should be further considered. While several phase I studies have reported data on safety and immunogenicity, there still lacks comprehensive translational evidence on the application of vaccines in routine clinical practice. Notably, the cited trials have explored heterogeneous mRNA vaccine targets, since they have included either patient-specific tumor neoantigens (personalized vaccines) or targets prearranged for research participants (e.g., the mRNA-5671/V941 vaccine targeting *KRAS*: G12D, G12V, G13D, G12C). Another issue is related to interventions themselves, as different trials have explored mRNA vaccines either in monotherapy or combined with other drugs (including pembrolizumab, atezolizumab, and mFOLFIRINOX), making it harder to extrapolate broader results. Large, multicenter and randomized clinical trials that encompass appropriate and well-structured control arms are still lacking, which is crucial to fully assess the safety and efficacy of mRNA vaccines. Additionally, the route of administration and the delivery system may influence clinical outcomes. Establishment of the most effective treatment modality remains a major concern for the success of mRNA vaccines in the context of PDAC.

**Figure 4:**
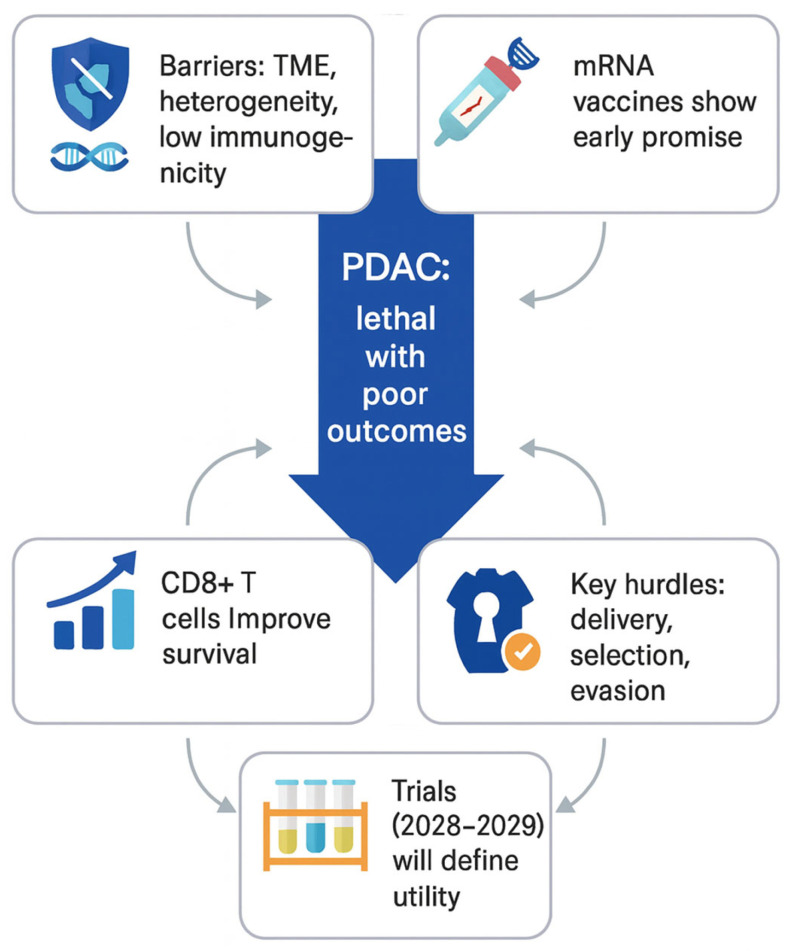
Key considerations for the development of personalized mRNA-based cancer vaccines in PDAC.

Altogether, mRNA vaccines might become a milestone in pancreatic cancer therapy; however, further extensive biomedical research, combined with evidence from published trials, focused on these limitations and challenges is essential. Figure 4 presents the key factors shaping the development of mRNA-based vaccines in PDAC management.

## 5. Materials and Methods

This review summarizes recent evidence on the role of mRNA vaccines in PDAC management. A literature search was performed in PubMed, PubMed Central, Scopus, and Google Scholar, with emphasis on the up-to-date publications and ongoing clinical trials from the ClinicalTrials.gov database. The search period for the sources included in the main part of this study covered the years 2020–2025. The search was based on a combination of keywords, such as “pancreatic ductal adenocarcinoma”, “mRNA vaccines”, “tumor neoantigens”, “tumor microenvironment”, “immune checkpoint inhibition”, “therapeutic resistance”, “personalized cancer treatment”, and related phrases in the field of PDAC. The selection criteria were based on a review of clinical trials, primarily from peer-reviewed studies, supplemented with web-based sources for ongoing trials without published results. Preclinical studies were included to outline the theoretical framework of PDAC research in order to ensure the scientific comprehensiveness. The collected data were critically evaluated to summarize established knowledge in the field, review ongoing clinical trials and their outcomes, and assess the potential clinical utility of mRNA-based therapies in PDAC.

## 6. Conclusions

Personalized mRNA vaccines targeting tumor neoantigens represent a highly promising and versatile strategy capable of overcoming key biological and immunological barriers inherent to PDAC. Nevertheless, most of the current evidence is derived from early-phase trials involving limited cohorts, and long-term clinical benefit remains to be confirmed. Robust conclusions regarding efficacy and survival impact will depend on the outcomes of ongoing randomized trials, many of which are not expected to report final results until 2028–2029. Continued advancement in neoantigen discovery, delivery technologies, and rational combination strategies will be essential for realizing the full therapeutic potential of mRNA-based cancer vaccines in PDAC.

## Figures and Tables

**Table 1:** Summary of clinical trials of mRNA vaccines in PDAC.

Vaccine Name	No. of Patients	Timeframe /Phase	Encoding Antigens	Main Action	Delivery System	Intervention	Administration Route	Results	Status
Personalized mRNA vaccine (NCT03468244)	24	2018–20 /Phase I	Patient- specific tumor neoantigens	T-cell stimulation	LPP	Personalized mRNA vaccine encoding neoantigens	Intramuscular	No serious adverse events; immune response in all patients	Finished
mRNA-5671/V941 (NCT03948763)	70	2019–22 /Phase I	*KRAS*: G12D, G12V, G13D, G12C	T-cell stimulation	LNP	mRNA-5671/V941 targeting KRAS mutations as monotherapy or in combination with pembrolizumab	Intramuscular	No published results	Finished
Autogene Cevumeran (NCT04161755)	16	2019–21 /Phase I	Personalized neoantigens (≤20)	T-cell stimulation	LPX	Autogene cevumeran (personalized mRNA vaccine) + atezolizumab + mFOLFIRINOX	Intravenous	Immune response in 50% of patients; no recurrence of disease for 18 months in responders	Finished
Autogene Cevumeran (NCT03289962)	272 (213)	2017–25 /Phase I	Personalized neoantigens (≤20)	T-cell stimulation	LPX	Autogene cevumeran (personalized mRNA vaccine) ± atezolizumab	Intravenous	Safety confirmed; immune response in patients with various solid tumors	Finished
Autogene Cevumeran (NCT05968326)	260	2023–29 /Phase II	Personalized neoantigens (≤20)	T-cell stimulation	LPX	Autogene cevumeran + atezolizumab + mFOLFIRINOX vs. mFOLFIRINOX	Intravenous	Recruiting	In progress
mRNA-0217/S001 (NCT05916261)	54	2023–25 /Phase I	Personalized neoantigens	T-cell stimulation	LNP	Personalized vaccine mRNA-0217/S001 ± pembrolizumab	Intramuscular	Recruiting	In progress
Personalized mRNA vaccine + adebrelimab (NCT06156267)	30	2024–26 /Phase I	Personalized neoantigens	T-cell stimulation	LNP	Personalized mRNA vaccine + adebrelimab	Not specified	Not yet recruiting	Planned
XH001 (NCT06353646)	24	2024–26 /Unknown	Personalized neoantigens	T-cell stimulation	Not specified	XH001 + ipilimumab + chemotherapy	Not specified	Recruiting	In progress
XP-004 (NCT06496373)	20	2024–27 /Unknown	Personalized neoantigens	T-cell stimulation	LNP	XP-004 + inhibitor	Not specified	Recruiting	In progress

Annotation. LNP—lipid nanoparticle; LPX—RNA-lipoplex; LPP—lipopolyplex; *KRAS*—Kirsten rat sarcoma virus.

## Data Availability

The datasets used and/or analyzed during the current study are available from the corresponding author upon reasonable request.
